# Analytical and numerical solutions of pore formation in elastic food materials during dehydration

**DOI:** 10.1016/j.crfs.2024.100762

**Published:** 2024-05-16

**Authors:** R.G.M. van der Sman, Michele Curatolo, Luciano Teresi

**Affiliations:** a Wageningen-Food & Biobased Research, Netherlands; b Food Process Engineering, Wageningen University & Research, Netherlands; c Roma Tre University, Italy

**Keywords:** Multiphysics, Large deformation mechanics, Drying, Simulation, Pore formation

## Abstract

In this paper, we describe a model for pore formation in food materials during drying. As a proxy for fruits and vegetables, we take a spherical hydrogel, with a stiff elastic skin, and a central cavity filled with air and water vapour. The model describes moisture transport coupled to large deformation mechanics. Both stress and chemical potential are derived from a free energy functional, following the framework developed by Suo and coworkers. We have compared Finite Volume and Finite Element implementations and analytical solutions with each other, and we show that they render similar solutions. The Finite Element solver has a larger range of numerical stability than the Finite Volume solver, and the analytical solution also has a limited range of validity. Since the Finite Element solver operates using the mathematically intricate weak form, we introduce the method in a tutorial manner for food scientists.

Subsequently, we have explored the physics of the pore formation problem further with the Finite Element solver. We show that the presence of an elastic skin is a prerequisite for the growth of the central cavity. The elastic skin must have an elastic modulus of at least 10 times that of the hydrogel. An initial pore with 10% of the size of the gel can grow to 5 times its initial size. Such an increase in porosity has been reported in the literature on drying of vegetables, if a dense hard skin is formed, known as case hardening. We discuss that models as presented in this paper, where moisture transport is strongly coupled to large deformation mechanics, are required if one wants to describe pore/structure formation during drying and intensive heating (as baking and frying) of food materials from first principles.

## Highlights

• Multiphysics model coupling large deformation and moisture transport is presented.
• Mechanistic explanation for pore formation during drying is provided.
• Skin formation is a prerequisite for pore formation.
• Tutorial introduction to weak formulation of large deformation mechanics is given.

## Graphical abstract

**Figure:**
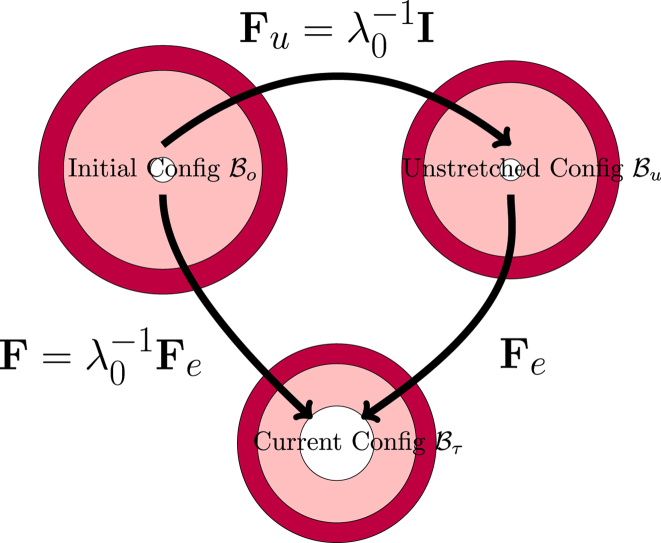

## 1 Introduction

In the last decade, researchers are starting to understand how large mechanical deformation is interacting with (de)hydrating vegetables (Aregawi et al., 2014; Karunasena et al., 2014; Liu et al., 2015; Singh et al., 2015; Gulati and Datta, 2015) (Van der Sman, 2015; Gulati et al., 2016; Mahbubur Rahman et al., 2018; Defraeye and Radu, 2018). For a quantitative understanding of this phenomenon, we think the multiphysics theory developed by Suo and coworkers is of seminal importance (Hong et al., 2008, 2009; Zhao et al., 2008; Marcombe et al., 2010). Their theory describes how to couple large deformation mechanics to moisture transport. Commonly, these multiphysics models are applied to the swelling of synthetic hydrogels, and the application of the theory to food materials is scarce (Van der Sman, 2015; Jin and van der Sman, 2022). The theory centers on a free energy function, which extends the Flory-Rehner theory but allows for inhomogeneous deformation. From the free energy functional the driving forces for momentum and mass transport are derived. As such the theory has been successful in describing phenomena as wrinkling (Weiss et al., 2013; Jin et al., 2015), which is especially extensive if the material has a stiff outer surface and soft inner part.

Another phenomenon due to the coupling of large deformations and dehydration is the development of pores during drying of polymeric food materials or vegetables (Maurice et al., 2017) (Jacob et al., 2016; Nguyen et al., 2018; Both et al., 2020; Siemons et al., 2020), (Zogzas et al., 1994; Karathanos et al., 1996; Khalloufi et al., 2015; Joardder et al., 2018). A requirement for this pore formation is the development of an elastic skin (Siemons et al., 2020), which can happen either that the skin forms a gel or that it enters the glassy state (Siemons et al., 2022). Further shrinkage of the drying food material, leads to buildup of elastic stresses in the skin, and underpressures in the core. Pre-existing pores, such as intercellular junctions in vegetables, will expand if the underpressure exceeds a critical value.

During vegetable drying, the formation of a rigid skin is called case hardening (Gulati and Datta, 2015). Some vegetables and fruits have a skin by nature, like cherries and plums. Especially, plums are known to form wrinkles during drying, which is taken as a prototypical example of wrinkling in soft matter sciences (Li et al., 2011) (Liu et al., 2015) (Genzer and Groenewold, 2006).

Such pore opening phenomena during drying have also been observed during drying of other soft porous materials (Nguyen et al., 2020). Such phenomena are investigated theoretically (Wang and Cai, 2015a, 2015b), where a constrained hydrogel with central cavity is subject to dehydration. If the outer skin is sufficiently rigid, growth of the central cavity is observed. Comparable physics are studied in (Curatolo et al., 2018) (Curatolo et al., 2023), which also regard a hydrogel with a central cavity subject to dehydration, but where the cavity remains filled with water.

Similar phenomena also play a role in biology. At excessive stresses, the liquid pressure in the xylem of vascular plants can become negative, leading to embolism, i.e. cavitation of gas bubbles (Konrad and Roth-Nebelsick, 2003) (Stroock et al., 2014). These conditions have been investigated experimentally using gels under tension (Vincent et al., 2014) (Wang et al., 2023). Cavitation and pore growth is a means to relax the tension (Vincent et al., 2014), but the growth can happen too fast under unstable conditions (Wang et al., 2023) (Sida et al., 2023). Gas bubbles can not exist under these conditions. It will grow unboundedly. In practice these conditions will lead to bursting or fracturing, rendering access of the bubble to the environment (Wang et al., 2023). This instability of gas-filled pores under tension is used by ferns for spores dispersal (Kang et al., 2017) (Kang et al., 2021). The physical description of this instability would involve accounting for inertia, and fracture properties of the biological tissue. However, such extreme mechanics are not observed during the drying of food materials.

In this paper, we examine the transient problem of pore development during dehydration using a simplified system, capturing the essentials of drying food materials. As an idealization of drying vegetable/fruit, we assume a sphere with an elastic skin and a core made of hydrogel. In the centre of the sphere is a small cavity, representing an intercellular pore. Hydrogel material is a good approximation for vegetable/fruit tissue, as our earlier study has shown that their water-holding capacity is well described by the classical Flory-Rehner theory, which is commonly applied to swelling of hydrogels (van der Sman et al., 2013).

In a previous paper we have developed for a similar problem a Finite Volume implementation of the model, where the large deformation mechanics is coupled to moisture transport, cf. (Bertrand et al., 2016; Jin and van der Sman, 2022). With this paper, we want to validate our Finite Volume approach via comparison to a Finite Element solution and an analytical approximation to the dehydrating pore/core/shell problem. Problems with coupled mechanics and moisture transport are commonly solved with the Finite Element method using the weak formulation. However, in the field of food science, the weak formulation is rarely used because of the involved mathematical technicalities. Hence, this paper will also have a strong tutorial character. Consequently, in the appendix, we give an elaborate introduction to the model description using the weak formulation.

The Finite Volume scheme is based on a reference frame, co-moving with the solid phase (polymer network). The Finite Element solution is based on a Lagrangian description of the problem, with the reference frame equal to the initial, stress-free free-swollen state, which is solved by COMSOL. Furthermore, we give an analytical approximated solution to this problem under the assumption of large pores and negligible gradients in the moisture content. After comparison of the three solutions, we will perform an extensive parameter study, whose results we use to discuss the physics of the problem. We conclude with a discussion of the advantages of the different approaches.

## 2 Theory

### 2.1 State variables & kinematics

In solid mechanics it is custom to describe the large deformation with respect to a non-moving reference frame $B_{\mathrm{ref}}$ (Hong et al., 2008). The position of a material point in this reference frame $B_{\mathrm{ref}}$ is given by **X**, but due to deformation the material point has moved to a new position in the current frame $\left(B_{\tau }\right)$: **x** = *f*(**X**, *t*) = **X** + **u**(**X**, *t*), with **u** the displacement vector. Via the deformation gradient tensor **F** = ∇*f* = **I** + ∇**u** (with **I** the identity tensor), one can compute how geometric elements transform from the reference frame $B_{\mathrm{ref}}$ to the current frame $B_{\tau }$. We consider the transformation of a volume element *dv*_*ref*_, a surface element *da*_*ref*_, and a normal vector **m** = **n**_*ref*_ from the reference frame $B_{\mathrm{ref}}$ to those (*dv*, *da*, **n**) in the current frame $B_{\tau }$:

(1) $$dv=J\;dv_{\mathrm{ref}},\;da=|F^{*}\;m|\;da_{\mathrm{ref}},\;n=\frac{F^{*}\;m}{\left|F^{*}\;m\right|}$$

with the Jacobian *J* = det **F**, and the conjugate F* = *J* **F**^−*T*^. Below, we will use these relations for transformations of the governing equations from the current frame to the reference frame. In solid mechanics it is custom to solve the problem in the reference frame. Also, we define the stretch parameters *λ*_*i*_, with $\lambda_{i}^{2}$ the eigenvalues of the Cauchy tensor **C** = **F**^*T*^**F**.

We like to note that in literature there is still a debate what is the correct reference state for polymer gels (Urayama et al., 1996) (Quesada-Pé et al., 2011) (Sakumichi et al., 2022). Often for synthetic polymers the dry state (without solvent) is taken as the reference state (Hong et al., 2009). However, in polymer physics, it is argued that the polymer fraction at preparation (with solvent present) must be taken as the reference configuration. Yet, food gels have often physical crosslinks, and consequently, there is no clearly defined preparation state.

Because we have a bilayer material (core-shell material), which is stress-free and uniformly swollen in the initial state, it can be shown that a dry configuration with zero stress and uniform deformation is physically not realizable. Hence, for our problem, it is more convenient to use the initial, free swollen configuration $B_{0}$ as the reference frame. The system attains its free swollen configuration in equilibrium with pure water, with water activity *a*_*w*_ = 1. In the free swollen configuration the system has uniform deformation and zero stress.

Yet, (food) hydrogels the biopolymers in the gel network are elastically stretched in the initial free swollen configuration state. Consequently, in polymer physics one takes as the reference frame the condition, where the biopolymers are unstretched. In the initial state the biopolymers have an uniform, isotropic stretch *λ*_*i*_ = *λ*_0_. In our earlier paper (RGM Van der Sman, 2015) we have shown that for many food materials there is a universal value $J_{0}=\lambda_{0}^{3}=3/2$, independent on the degree of crosslinking. There we have defined the polymer volume fraction in the free-swollen configuration as *φ*_0_, and the polymer volume fraction in the unstretched configuration as $\varphi_{\mathrm{ref}}=\lambda_{0}^{3}\varphi_{0}$.

As our system is a bilayer core/shell system with each layer having different crosslink density, we think it is convenient to define the deformation with the respect to take the initial free-swollen configuration $B_{0}$, but define the elastic deformation with respect to the unstretched configuration $B_{u}$. Hence, the elastic deformation **F**_*e*_ is defined following a multiplicative decomposition of **F**:

(2) $$F=F_{e}F_{u}$$

Thus in the initial state **F**=**I**, and **F**_*e*_ = *λ*_0_**I**, and thus it follows that $F_{u}=\lambda_{0}^{-1}I$.

**Fig. 1:**
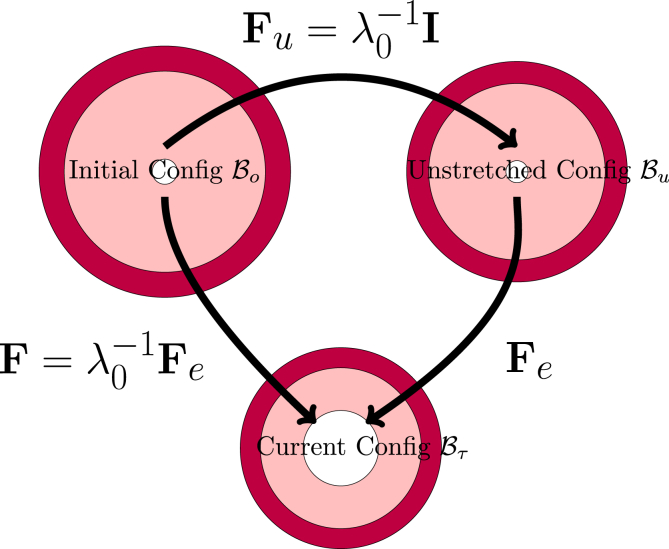
Definition of the various configurations for defining the deformation. The initial configuration is used as the domain frame, defining the deformation **F** to the current (deformed) configuration. The elastic deformation **F**_*e*_ is defined via the reference configuration. Note, that in all 3 configurations, the skin and core have matching deformations at their interface.

A similar multiplicative decomposition of **F** is commonplace in the field of plastic deformation, where the total deformation has both inelastic (plastic) and elastic contributions, with the latter defined with respect to an intermediate stress-free configuration (Simo, 1988). In our problem, the unstretch configuration frame serves as our intermediate configuration, from which we compute the elastic deformation. Note, that core and shell have different unstretched reference configurations, as they differ in *φ*_*ref*_. This multiplicative decomposition and its connection to the three defined coordinate frames is nicely illustrated in Fig. 1. Thus, we have the following transformations.

● **F** maps the deformation of material points from the initial configuration $B_{0}$ to the current configuration $B_{\tau }$, with det **F** = *J* = *φ*_0_/*φ*,
● **F**_*u*_ maps from the initial configuration $B_{0}$ to the intermediate unstretched configuration $B_{u}$, with det **F**_*u*_ = *J*_*u*_ = *φ*_0_/*φ*_*ref*_, and
● the elastic deformation **F**_*e*_ maps from the intermediate unstretched configuration $B_{u}$ to the current configuration $B_{\tau }$, with $\det F_{e}=J_{e}=1/\tilde{\varphi }=\varphi_{\mathrm{ref}}/\varphi$,

Thus, instead of the immediate mapping **F**, one can do this also in two steps: via **F**_*u*_, and **F**_*e*_. The stress will be a function of the elastic deformation, which will be computed as: $F_{e}=FF_{u}^{-1}=FF_{0}=\lambda_{0}F$. Note, $\tilde{\varphi }$ we have used earlier in our papers about Flory-Rehner theory (RGM Van der Sman, 2015).

#### 2.1.1 Spherical symmetry

Under the assumptions of spherical symmetry, a material point *X* is uniquely determined by the radial coordinate *R*, and the only non trivial component of the displacement **u** is the radial displacement *u* = *u*(*R*, *t*); the current position *x* is represented by *r* = *R* + *u*(*R*, *t*). The deformation gradient **F** is given by:

(3) $$F=\left(\begin{array}{ccc} \lambda_{r} & 0 & 0\\ 0 & \lambda_{\theta } & 0\\ 0 & 0 & \lambda_{\theta } \end{array}\right),\;J=\lambda_{r}\;\lambda_{\theta }^{2},\;with\;\lambda_{r}=1+\frac{du}{dR},\;\lambda_{\theta }=1+\frac{u}{R},$$

where *λ*_*r*_, *λ*_*θ*_ are the radial and the hoop stretches measured with respect to the free swollen configuration $B_{o}$. Also the composition of deformations and their determinants simplifies as:

(4) $$F_{e}=F\;F_{o}\;\Rightarrow \;\lambda_{e,r}=\lambda_{r}\;\lambda_{o},\;\lambda_{e,\theta }=\lambda_{\theta }\;\lambda_{o},\;J_{e}=\lambda_{r}\;\lambda_{\theta }^{2}\;\lambda_{o}^{3}.$$

### 2.2 Balance laws & constitutive relations

In this section, we describe the model for the osmotic dehydration and deformation of a spherical hydrogel, with a gas-filled cavity, and a permeable elastic skin. The elastic skin can also absorb water, but it has a higher crosslink density than the core. Hence, the two domains of our core-shell system have the following reference values (*φ*_*ref*_):

(5) $$\varphi_{\mathrm{core}}=0.05,\;\varphi_{\mathrm{skin}}=\varphi_{\mathrm{core}}\;{\left(\frac{G_{\mathrm{skin}}}{G_{\mathrm{core}}}\right)}^{1/\beta },\;\text{with }\beta =\frac{9}{4},$$

where *G*_*skin*_ and *G*_*core*_ is the shear modulus of the two regions. The value of *φ*_*skin*_ follows from the *c*∗-theorem of deGennes (RGM Van der Sman, 2015).

#### 2.2.1 Mass balance of water

First, we describe the balance equation of liquid in the current frame, that is using the spatial fields as *φ*_*w*_ defined on $B_{\tau }$. Let *D*_*t*_ = *∂*_*t*_ + ∇ ⋅**v** be the material derivative, with **v** the spatial velocity defined by the time derivative of the deformation field $\dot{u}$. The mass balance for the water in the core and gel is:

(6) $$D_{t}\;\varphi_{w}=-\nabla \cdot j_{w}=\nabla \cdot \frac{D_{s}\;\nu_{w}}{R_{\mathrm{gas}}\;T}\;\nabla \mu_{w},\;\text{on }B_{\tau },$$

where **j**_*w*_ is the diffusive water flux given by the generalized Darcy's law, *D*_*s*_ is the self-diffusivity of water, and *μ*_*w*_ is the chemical potential (in units of [*J*/*m*^3^]).

The chemical potential is derived from a free energy functional (Hong et al., 2009). It can be decomposed into two contributions:

(7) $$\mu_{w}=\mu_{w,mix}+p_{\mathrm{liq}}=-\Pi_{\mathrm{mix}}+p_{\mathrm{liq}}$$

*p*_*liq*_ is the hydrostatic pressure in the solvent, which is a consequence of the incompressibility of the hydrogel material. *μ*_*w*,*mix*_ is the so-called mixing contribution, and it can be derived from Flory-Huggins theory for polymer gels.

However, it is convenient to reformulate the mixing contribution into an osmotic pressure Π_*mix*_, which follows the scaling law of Cloizeaux (RGM Van der Sman, 2015). Earlier, we have shown that the response of food gel materials under osmotic pressure can be mapped to a single master curve (RGM Van der Sman, 2015), which is a consequence of the c* theorem of deGennes (RGM Van der Sman, 2015). From this scaling behaviour it turned out that the mixing pressure can be reformulated in terms of the elastic modulus of the material *G*:

(8) $$\Pi_{\mathrm{mix}}=\alpha \;G\;{\tilde{\varphi }}^{\beta },\;\text{with }\beta =\frac{9}{4},\;\tilde{\varphi }=\frac{\varphi_{s}}{\varphi_{\mathrm{ref}}}$$

and *α* is a constant, defined in (RGM Van der Sman, 2015).

#### 2.2.2 Balance of forces on $B_{\tau }$

We assume that inertia forces are negligible and external forces are null; the balance of forces in the current configuration writes as:

(9) $$\nabla \cdot \sigma =0,\;\text{on }B_{\tau },\;\sigma \;n=t,\;\text{on }\partial B_{\tau },$$

where *σ* is the actual stress (Cauchy stress), **n** the normal to the boundary $\partial B_{\tau }$, and **t** the force at boundary. Under spherical symmetry hypothesis, the non zero components of the stress are *σ*_*rr*_, and *σ*_*θθ*_.

By assuming the Neo-Hookean model, and setting $\hat{\varphi }=\varphi /\varphi_{0}\lambda_{0}$, the stress components are given by (Van der Sman, 2015):

(10) $$\sigma_{i}=\tilde{\varphi }\;G\;\lambda_{e,i}^{2}-p=\frac{\varphi }{\varphi_{0}}\frac{\lambda_{0}^{2}}{\lambda_{0}^{3}}\;G\;\lambda_{i}^{2}-p\;\Rightarrow \;\sigma_{i}=\hat{\varphi }\;G\;\lambda_{i}^{2}-p$$

Using $\varphi /\varphi_{0}=1/\lambda_{\theta }^{2}\lambda_{r}$ the stress components are:

(11) $$\sigma_{rr}=G\;\frac{\lambda_{r}}{\lambda_{\theta }^{2}\;\lambda_{0}}-p_{\mathrm{liq}},\;\sigma_{\theta \theta }=G\;\frac{1}{\lambda_{r}\;\lambda_{0}}-p_{\mathrm{liq}}$$

From (9), it follows:

(12) $$\partial_{r}\sigma_{rr}+\frac{2}{r}\left(\sigma_{rr}-\sigma_{\theta \theta }\right)=0$$

From the difference between radial and hoop stresses, we can eliminate the pressure:

(13) $$\sigma_{rr}-\sigma_{\theta \theta }=\hat{\varphi }\;G\;\left(\lambda_{r}^{2}-\lambda_{\theta }^{2}\right)$$

The condition for mechanical equilibrium becomes:

(14) $$\partial_{r}\sigma_{rr}=-\frac{2G}{r}\hat{\varphi }\;\left(\lambda_{r}^{2}-\lambda_{\theta }^{2}\right)$$

This equation will be integrated numerically to obtain *σ*_*rr*_(*r*). Subsequently, we can compute the pressure field *p*_*liq*_ via:

(15) $$p_{\mathrm{liq}}\left(r\right)=G\;\hat{\varphi }\;\lambda_{r}^{2}\left(r\right)-\sigma_{rr}\left(r\right)$$

which can be inserted in the definition of the water chemical potential, which enters the mass flux **j**_*w*_. The problem is complemented by the boundary conditions. At the outer boundary the stress balances the external pressure *p*_0_:

(16) $$\sigma_{rr}\left(r=r_{\mathrm{out}}\right)=-p_{0}$$

Note, by convention the stress and pressure have opposite signs. At the inner boundary at the wall of the cavity holds:

(17) $$\sigma_{rr}\left(r=r_{in}\right)=-p_{\mathrm{gas}}$$

with *p*_*gas*_ the gas pressure in the central cavity, which will be derived below. The boundary condition for the chemical potential at the outer boundary is

(18) $$\mu_{w}\left(r=r_{\mathrm{out}}\right)=p_{0}-\Pi_{\mathrm{ext}}=\mu_{w,ext}$$

Π_*ext*_ is the osmotic pressure of the external fluid.

The central cavity is assumed to be filled with both air (inert nitrogen) and water vapour. We assume that the air does not dissolve in the hydrogel, but the water vapour can be exchanged with the hydrogel.

We assume local equilibrium for the gas bubble in the cavity. In the free-swollen initial state it holds that *p*_*gas*_ = *p*_0_, and the vapour in the cavity is saturated (*a*_*w*_ = 1)*p*_*vap,0*_ = *p*_*sat*_(*T*). Thus the amount of air is *p*_*air,0*_ = *p*_0_ − *p*_*vap,0*_, and *N*_*air,0*_ = *p*_*air,0*_*V*_*gas,0*_/*RT*, with the initial gas volume $V_{\mathrm{gas,0}}=4\pi r_{0}^{3}/3$. This amount of air, *N*_*air,0*_ = *N*_*air*_ remains constant throughout the drying.

The equilibrium condition for the cavity is:

(19) $$\mu_{w,gas}=\frac{R_{\mathrm{gas}}T}{\nu_{w}}\log\left(\frac{p_{\mathrm{vap}}}{p_{\mathrm{sat}}\left(T\right)}\right)+p_{\mathrm{gas}}=\mu_{w}\left(r=r_{in}\right)$$

with *p*_*gas*_ = *p*_*air*_ + *p*_*vap*_, and *p*_*air*_ = *N*_*air*_*RT*/*V*_*gas*_, using $V_{\mathrm{gas}}=4\pi r_{in}^{3}/3$ for the gas bubble volume.

### 2.3 Finite Volume solution

#### 2.3.1 Transient solution

With the Finite Volume we solve the problem in the current frame $B_{\tau }$, which moves along with the biopolymer network. The (current) domain is subdivided into control volumes being spherical shells, with same thickness smaller than the outer radius: Δ*r* ≪ *r*_*out*_. The boundaries of the different domains, i.e. a) the external surface, b) the internal surface of the cavity, and c) the interface between core and shell, exactly match with the boundary between control volumes. The state variable *φ*_*w*_ represents the volume-averaged value of the control volume and approximates the value at the centre of the control volume. The coordinate system will evolve with the dimensional changes of the control volumes, as follows from the temporal evolution of *φ*_*w*_. The latter will be driven by gradients in *μ*_*w*_, which are also computed at the centers of the control volumes. However, the fluxes *J*_*w*,*r*_ are computed at the boundaries of the control volumes following central differencing:

(20) $$J_{w,r}\left(r\right)\approx -\frac{D_{m}\nu_{w}}{R_{\mathrm{gas}}T}\frac{\mu_{w}\left(r+\Delta r/2\right)-\mu_{w}\left(r-\Delta r/2\right)}{\Delta r}4\pi r^{2}$$

with Δ*r* the distance between centers of the adjacent control volumes. With the fluxes one can compute the change in the volume of water inside the control volume, using simple Euler forward time integration:

(21) $$\begin{array}{lll} & & \frac{\Delta V_{w}\left(r,t+\Delta t\right)-\Delta V_{w}\left(r,t\right)}{\Delta t}=\\ & & \text{Jw,r}(\text{r}+\Delta \text{r}+/2)-\text{Jw,r}(\text{r}-\Delta \text{r}-/2) \end{array}$$

with Δ*r*^+^ and Δ*r*^−^ distances to adjacent control volumes. Note, that the volume of the polymers Δ*V*_*s*_ in the control volume always stays the same, as the coordinate system is linked to the deforming polymer network. The polymer volume fraction then reads:

(22) $$\varphi_{w}\left(r\right)=\frac{\Delta V_{w}\left(r\right)}{\Delta V_{w}\left(r\right)+\Delta V_{s}\left(r\right)}$$

Mind, that the chemical potential contains the hydrostatic pressure *p*_*liq*_, as *μ*_*w*_ = −Π_*mix*_(*φ*_*s*_) + *p*_*liq*_. *p*_*liq*_ follows from solving the momentum balance: ∇ ⋅ *σ* = 0. This equation will be integrated starting at the outside *r* = *r*_*out*_. The boundary condition states *σ*_*rr*_(*r* = *r*_*out*_) = −*p*_0_. The radial stress will be computed at the boundaries of the control volumes (with Δ*r* the thickness of the control volume):

(23) $$\sigma_{rr}\left(r-\Delta r/2\right)=\sigma_{rr}\left(r+\Delta r/2\right)-\Delta r\frac{d\sigma_{rr}}{dr}\left(r\right)$$

with the gradient in the radial stress at the centre of the control volume:

(24) $$\frac{d\sigma_{rr}}{dr}\left(r\right)\approx \frac{2G\left(r\right)}{r}\hat{\varphi }\left(r\right)\;\left[\lambda_{\theta }^{2}\left(r\right)-\lambda_{r}^{2}\left(r\right)\right]$$

using this gradient we also compute *σ*_*rr*_(*r*), via which we compute the hydrostatic pressure:

(25) $$p_{\mathrm{liq}}\left(r\right)=G\left(r\right)\hat{\varphi }\left(r\right)\;\lambda_{r}^{2}\left(r\right)-\sigma_{rr}\left(r\right)$$

This pressure is substituted in *μ*_*w*_(*r*).

Via integration of the force balance follows the radial stress at the inner surface of the cavity: *σ*_*rr*_(*r*_*in*_) = −*p*_*gas*_, determining the gas pressure, and the chemical potential *μ*_*w*_(*r*_*in*_) = −*R*_*gas*_*T* log(*a*_*w*,*gas*_) + *p*_*gas*_. Thus follows the vapour pressure: *p*_*vap*_ = *a*_*w*,*gas*_*p*_*sat*_(*T*), and the air pressure *p*_*air*_ = *p*_*gas*_ − *p*_*vap*_. Subsequently, we compute the gas volume: *p*_*air*_*V*_*gas*_ = *p*_*air,0*_*V*_*gas,0*_. This finally renders the amount vapour moles in the cavity: *N*_*vap*_(*t* + Δ*t*) = *p*_*vap*_*V*_*gas*_/*R*_*gas*_*T*. We use this to compute the molar flux at the inner boundary:

(26) $$J_{w}\left(r_{in},t+\Delta t\right)=\frac{N_{\mathrm{vap}}\left(t+\Delta t\right)-N_{\mathrm{vap}}\left(t\right)}{\Delta t}$$

As the new gas volume is known, *V*_*gas*_ together with volumes of water, Δ*V*_*w*_(*r*), one can compute the new positions of the boundaries (*r* ± Δ*r*/2) and centers of the control volumes (*r*). We note that *J*_*w*_(*r*_*in*_) represents the amount of water turned into water vapour via evaporation. As we assumed isothermal conditions, the evaporation does not lead to evaporative cooling.

**Fig. 2:**
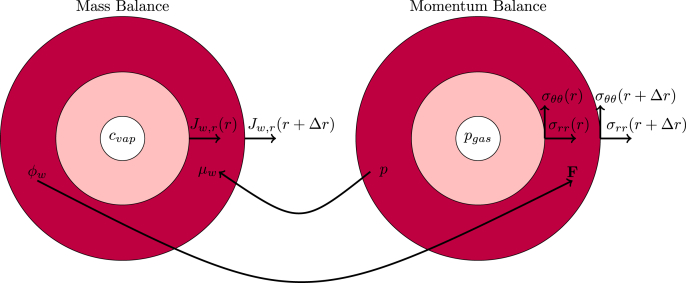
Schematic representation of the computation of mass and momentum balance, as computed via the Finite Volume methods. Details about the coupling between the balances is explained in the text.

In Fig. 2 we have indicated schematically how the mass and momentum balances are solved, and the coupling between them. The divergence of the mass fluxes *J*_*w*_ leads to changes in the amount of water (*φ*_*w*_), which leads to deformation of the sphere - as captured by the deformation gradient **F**=**I** + ∇**u**. The deformation leads to stresses *σ*_*ii*_, which can be viewed as momentum fluxes. The incompressibility constraint leads to the hydrostatic pressure *p*_*liq*_, which couples back to the chemical potential *μ*_*w*_, driving the mass fluxes.

#### 2.3.2 Steady state solution

We will also solve the steady state problem numerically, cf. (Van der Sman, 2015). As a first step, we integrate the momentum balance, given the external osmotic pressure Π_*ext*_, and an assumed stretch Λ_*b*_, which renders *p*_*gas*_, and Λ_*a*_ (or equivalently *V*_*gas*_). Local values of $\tilde{\varphi }$ for skin and gel is calculated from the chemical equilibrium: *μ*_*w*_(*r*) = *μ*_*w*,*ext*_, or rather Π_*mix*_ − *p*_*liq*_ + *p*_0_ = Π_*ext*_. From the solution of the momentum balance we compute *p*_*vap*_, via *p*_*gas*_ = *p*_*air*_ + *p*_*vap*_, and *p*_*air*_ = *N*_*air,0*_*R*_*gas*_*T*/*V*_*gas*_. This value of *p*_*vap*_ should match that of chemical equilibrium: *p*_*vap*_ = *p*_*sat*_(*T*) exp[(*μ*_*w*,*ext*_ − *p*_*gas*_)*ν*_*w*_/*R*_*gas*_*T*]. This non-linear equation is solved via the secant method.

### 2.4 Analytical solution

For the steady state solution we can formulate an analytical approximation, cf. (Van der Sman, 2015). Using the incompressibility condition, and defining *λ* = *λ*_*θ*_, the condition for mechanical equilibrium can be rewritten as:

(27) $$d\sigma_{rr}=2G\;\left(\hat{\varphi }\lambda^{2}-\frac{1}{\hat{\varphi }\lambda^{4}}\right)\frac{dr}{r}$$

Following Pence and Tsai (2006) (Van der Sman, 2015) we change the integration variable using the total derivative of *λ* = *r*/*R*:

(28) $$\frac{dr}{r}=\frac{d\lambda }{\lambda \left(\hat{\varphi }-\lambda^{3}\right)}$$

and consequently

(29) $$d\sigma_{rr}=2G\;\left(\hat{\varphi }\lambda^{2}-\frac{1}{\hat{\varphi }\lambda^{4}}\right)\frac{1}{\lambda -\hat{\varphi }\lambda^{4}}\;d\lambda$$

As a first approximation, we can assume that for thin to moderately thick shells, with *r*_*a*_ < *r* < *r*_*b*_, the swelling is uniform cf. (Pence and Tsai, 2006). For a homogeneous shell of hydrogel material with elastic modulus *G*, the integration of the condition for mechanical equilibrium gives the following analytical expression for the pressure drop Δ*p* over the shell:

(30) $$\frac{2\left(\Delta p\right)}{G}=\frac{1+4\hat{\varphi }\Lambda_{a}^{3}}{\hat{\varphi }\Lambda_{a}^{4}}-\frac{1+4\hat{\varphi }\Lambda_{b}^{3}}{\hat{\varphi }\Lambda_{b}^{4}}$$

with Λ_*a*_ = *λ*(*r* = *r*_*a*_), and Λ_*b*_ = *λ*(*r* = *r*_*b*_) the stretch factor at respectively the inside and outside of the shell. Note that, the above expression is mathematically identical to that derived in (Curatolo et al., 2018), although the dry state is used as the reference state.

An approximation for $\hat{\varphi }$ follows from the boundary condition at the outer boundary:

(31) $$G\hat{\varphi }\Lambda_{b}^{2}+\Pi_{\mathrm{ext}}-\Pi_{\mathrm{mix}}=0$$

Given the swelling Λ_*b*_ and the swelling ratio from the above relation, we can easily compute the inner radius *r*_*a*_ of the shell from the incompressibility condition:

(32) $$\varphi \left(r_{b}^{3}-r_{a}^{3}\right)=\varphi_{0}\left(R_{B}^{3}-R_{A}^{3}\right)$$

with *R*_*B*_ and *R*_*A*_ the dimensions of the shell in the initial state.

In our problem, we have a hydrogel with a core-shell structure, with elastic moduli *G*_*core*_ and *G*_*skin*_. The above expression for the pressure drop over a spherical shell can be applied to both core and shell. Hence, the pressure difference between the cavity and the environment can be decomposed in two contributions:

(33) $$p_{\mathrm{gas}}-p_{0}=\Delta p_{\mathrm{skin}}+\Delta p_{\mathrm{core}}$$

with

(34) $$\begin{array}{lll} \Delta p_{\mathrm{skin}} & = & \frac{G_{\mathrm{skin}}}{2}\left[\frac{1+4{\tilde{\varphi }}_{\mathrm{skin}}\Lambda_{m}^{3}}{{\hat{\varphi }}_{\mathrm{skin}}\Lambda_{m}^{4}}-\frac{1+4{\hat{\varphi }}_{\mathrm{skin}}\Lambda_{\mathrm{out}}^{3}}{{\hat{\varphi }}_{\mathrm{skin}}\Lambda_{\mathrm{out}}^{4}}\right]\\ \Delta p_{\mathrm{core}} & = & \frac{G_{\mathrm{core}}}{2}\left[\frac{1+4{\hat{\varphi }}_{\mathrm{core}}\Lambda_{in}^{3}}{{\hat{\varphi }}_{\mathrm{core}}\Lambda_{in}^{4}}-\frac{1+4{\hat{\varphi }}_{\mathrm{core}}\Lambda_{m}^{3}}{{\hat{\varphi }}_{\mathrm{core}}\Lambda_{m}^{4}}\right] \end{array}$$

Λ_*m*_ is the stretch factor at the interface between core and shell (*r* = *r*_*m*_), Λ_*in*_ and Λ_*out*_ are the stretch factors at *r*_*in*_ and *r*_*out*_ respectively. ${\hat{\varphi }}_{\mathrm{skin}}$ follows from the chemical equilibrium at *r* = *r*_*out*_, and ${\hat{\varphi }}_{\mathrm{core}}$ follows from the chemical equilibrium at *r* = *r*_*m*_, using that *p*_*liq*_(*r* = *r*_*m*_) = *p*_0_ + Δ*p*_*skin*_. Note, that in steady state at every location, the chemical potential equals that of the environment: *μ*_*w*_(*r*) = *μ*_*w*,*ext*_

### 2.5 Finite Element solution using weak formulation

Following the common approach in solid mechanics for hydrogels (Hong et al., 2008), we solve the problem also via the Finite Element method using the weak formulation with the initial frame $B_{o}$ as the reference configuration. As researchers in food science are often not familiar with the weak form, we give a short explanation how to derive it in the Appendix. Before stating the weak formulations, we reformulate the mass and force balances in terms of the spatial fields defined in the initial frame $B_{o}$.

#### 2.5.1 Mass balance of water on $B_{o}$

Above, the mass balance of water has been written on the current configuration $B_{\tau }$ by using as state variable the volume fraction *φ*_*w*_, see (6). In Finite Element models one uses as the state variable for the mass balance the molar concentration *c* of water per volume in the initial frame $B_{o}$ (Hong et al., 2009). This relates to the volume change *J* due to deformation:

(35) $$J=\lambda_{r}\;\lambda_{\theta }^{2}=\hat{J}=\varphi_{0}+\nu_{w}\;c,$$

where *ν*_*w*_ is the water molar-volume. Note, that the volume fraction of water in the current frame $B_{\tau }$ equals *φ*_*w*_ = 1 − *φ*_*s*_ = 1 − *φ*. Thus, *φ*_*w*_ = *ν*_*w*_*c*/*J*. Hence, the state variable *c* also accounts for volume changes due to dehydration, i.e. it is linear with the pull-back of the volume fraction *φ*_*w*_, and it might be used for the incompressibility condition.

The mass balance (6) written on $B_{\tau }$, can be reformulated in the reference frame $B_{o}$ as follows:

(36) $$\partial_{t}c=-\nabla \cdot h_{w},\;\text{on }B_{o},\;h_{w}\cdot m=q,\;\text{on }\partial B_{o},$$

where **h**_*w*_ is the pull-back of the current molar flux **j**_*w*_/*ν*_*w*_, **m** the normal to the boundary $\partial B_{o}$, and *q* the liquid flux at boundary. From the formula relating the spatial gradient ∇*μ*_*w*_(*x*, *t*) to the material gradient ∇*μ*_*w*_(*X*, *t*):

(37) $$\nabla \mu_{w}\left(x,t\right)=F{\left(X,t\right)}^{-T}\;\nabla_{X}\mu_{w}\left(X,t\right)$$

it follows:

(38) $$h_{w}=\frac{1}{\nu_{w}}\;J\;F^{-1}\;j_{w}=J\;F^{-1}\;\frac{D_{s}}{R_{\mathrm{gas}}\;T}\;F^{-T}\;\nabla \mu_{w}=\tilde{M}\;\nabla \mu_{w}.$$

The mobility tensor $\tilde{M}$ is given by

(39) $$\tilde{M}=J\;F^{-1}\;M\;F^{*},\;\text{with }M=\frac{D_{s}}{R_{\mathrm{gas}}\;T}.$$

with *M* the mobility in the current frame.

In spherical coordinates, the mass balance thus reads:

(40) $$\partial_{t}c=-\partial_{R}\frac{D_{s}}{RT}\;\frac{\lambda_{\theta }^{2}}{\lambda_{r}}\;\partial_{R}\mu_{w}.$$

The prefactor $\lambda_{\theta }^{2}/\lambda_{r}$ accounts for the deformation of the exchange surface area $\left(∼\lambda_{\theta }^{2}∼{\left(r/r_{0}\right)}^{2}\right)$ and the deformation of the thickness of control volumes: (∼*λ*_*r*_ ∼ (*dr*/*dr*_0_)).

#### 2.5.2 Balance of forces on $B_{o}$

The balance equation (9) written in the current frame $B_{\tau }$ can be reformulated in the reference frame $B_{o}$ as follows:

(41) $$\nabla \cdot S=0\;\text{on }B_{o},\;S\;m=t_{o},\;\text{on }\partial B_{o},$$

where **S** = *σ* F* is the reference stress (first Piola stress), and **t**_*o*_ = |F* **m**| **t** the force at boundary. Under the spherical symmetry hypothesis, using Eq. (11), the first Piola stress **S** simplifies as:

(42) $$S_{RR}=G\;\frac{\lambda_{r}}{\lambda_{0}}-p\;\lambda_{\theta }^{2},\;S_{\theta \theta }=G\;\frac{\lambda_{\theta }}{\lambda_{0}}-p\;\lambda_{\theta }\;\lambda_{r}.$$

The balance of forces (41) rewrites as

(43) $$\partial_{R}S_{RR}+\frac{2}{R}\left(S_{RR}+S_{\theta \theta }\right)=0.$$

#### 2.5.3 Weak formulation

We formulate the problem in the weak form. How to derive the weak forms is shown in the Appendix. We give only the left-hand side of the weak form, as the right-hand side is handled via the boundary conditions. The weak contribution *W*_1_ for the balance of forces reads:

(44) $$W_{1}\left(\tilde{u},\tilde{p}\right)=\int_{R_{in}}^{R_{\mathrm{out}}}-\left[S_{RR}\;\left(\partial_{R}\tilde{u}\right)+\frac{2}{R}\;S_{\theta \theta }\;\tilde{u}\right]\;4\;\pi \;R^{2}\;dR+{\left[\;\bar{p}\;\tilde{u}\;4\;\pi \;R^{2}\;\right]}_{\partial B_{o}},$$

where $\tilde{u}$ and $\tilde{p}$ are the test functions associated to the displacement *u* = *r* − *R*, and to the Lagrange multiplier *p*, respectively, and $\bar{p}$ is the effective pressure at the boundary. Note, that different relations hold for **S** in core and skin, as these layers differ in their elastic modulus *G*.

At the boundary we have:

(45) $$\begin{array}{lll} S_{RR}=-p_{o}\lambda_{\theta }^{2},\; & & \text{at }R=R_{\mathrm{out}}\;\left(\text{external}\right)\text{,} \end{array}$$

(46) $$\begin{array}{lll} S_{RR}=p_{\mathrm{gas}}\lambda_{\theta }^{2},\; & & \text{at }R=R_{in}\;\left(\text{cavity}\right). \end{array}$$

Their weak formulation is:

(47) $$\text{at }R=R_{\mathrm{out}}:\;\bar{p}=p_{0}\;\lambda_{\theta }^{2},\;\text{at }R=R_{in}:\;\hat{p}=-p_{\mathrm{gas}}\;\lambda_{\theta }^{2}.$$

The initial conditions are formulated for the displacement. As we use the initial state as the domain configuration, it follows that *u* = 0.

The weak contribution *W*_2_ for the incompressibility condition, where the pressure is represented in terms of a Lagrangian multiplier, reads:

(48) $$W_{2}\left(\tilde{u},\tilde{p}\right)=\int_{R_{in}}^{R_{\mathrm{out}}}\left(J-\hat{J}\right)\tilde{p}\;4\;\pi \;R^{2}\;dR,$$

with $J=\lambda_{r}\;\lambda_{\theta }^{2}$, and $\hat{J}=\nu_{w}c+\varphi_{0}$. Note, that $\varphi_{0}=\varphi_{\mathrm{ref}}/\lambda_{0}^{3}$ is different for core and skin. No boundary conditions are required for the incompressibility condition. The initial condition for the pressure field for the free-swollen state is different for the core and shell:

(49) $$\begin{array}{lll} p\left(t=0\right) & = & \frac{G_{\mathrm{core}}}{\lambda_{0}}+p_{0}\\ p\left(t=0\right) & = & \frac{G_{\mathrm{skin}}}{\lambda_{0}}+p_{0} \end{array}$$

The weak contribution to the mass balance is split into the core contribution *W*_*c*_ and the skin contribution *W*_*s*_:

(50) $$\begin{array}{lll} W_{c}\left({\tilde{c}}_{c}\right) & = & \int_{R_{in}}^{R_{m}}\left[{\tilde{c}}_{c}\;\partial_{t}c_{c}-h_{w,R}\;\partial_{R}\;{\tilde{c}}_{c}\right]\;4\;\pi \;R^{2}\;dR+{\left[q\;{\tilde{c}}_{c}\;4\;\pi \;R^{2}\right]}_{\partial \left(R_{in},R_{m}\right)}, \end{array}$$

(51) $$\begin{array}{lll} W_{s}\left({\tilde{c}}_{s}\right) & = & \int_{R_{m}}^{R_{\mathrm{out}}}\left[{\tilde{c}}_{s}\;\partial_{t}c_{s}-h_{w,R}\;\partial_{R}\;{\tilde{c}}_{s}\right]\;4\;\pi \;R^{2}\;dR,+{\left[q\;{\tilde{c}}_{s}\;4\;\pi \;R^{2}\right]}_{\partial \left(R_{m},R_{\mathrm{out}}\right)}, \end{array}$$

where *R*_*m*_ is the position of the interface core/skin at initial conditions, *q* the mass flux at the boundaries of the two subdomains. *c*_*s*_ and *c*_*c*_ are the state variable for water content in the skin and core respectively. Finally, *h*_*w*,*R*_ is the radial component of the flux **h**_*w*_ given by:

(52) $$h_{w,R}=-\frac{\lambda_{\theta }^{2}}{\lambda_{r}}\;\frac{D}{R_{\mathrm{gas}}\;T}\;\partial_{R}\mu_{w}.$$

The boundary conditions are formulated as Robin type relation for the molar fluxes. These conditions are different from the above Finite Volume model, where Dirichlet conditions are assumed for *μ*_*w*_. As the chemical potential is not a state variable in our Finite Element formulation, we found it more convenient to formulate it as a Robin-type boundary condition for the molar fluxes. Moreover, they allow a more gradual adaptation of the core/shell system to the environmental conditions. To ensure continuity of the chemical potential at the interface of core and shell (at *R*_*m*_), there a Robin-type boundary condition with the flux *q* linear in the difference between the chemical potentials at either side of the interface.

We assume convective boundary layers in the gas directly surrounding the skin and in the central cavity, allowing for evaporation of liquid water into vapour, and we assign different boundary conditions for the core and the skin; for the core we have:

(53) $$\begin{array}{ccc} q & =J_{\mathrm{cav}}=\lambda_{\theta }^{2}\;\beta_{\mathrm{cav}}\;\left(a_{w,int}-RH_{\mathrm{cav}}\right)\;c_{\mathrm{sat}}\left(T\right), & \text{at }R=R_{in}\;\left(\text{cavity}\right)\text{,}\\ q & =-D_{\mu }\;\left(\mu_{w,c}-\mu_{w,s}\right), & \text{at }R=R_{m}\;\left(\text{interface core/skin}\right)\text{,} \end{array}$$

for the skin

(54) $$\begin{array}{ccc} q & =D_{\mu }\;\left(\mu_{w,c}-\mu_{w,s}\right), & \text{at }R=R_{m}\;\left(\text{interface core/skin}\right)\text{,}\\ q & =J_{\mathrm{evap}}=\lambda_{\theta }^{2}\;\beta_{\mathrm{air}}\;\left(a_{w,ext}-RH_{\mathrm{ext}}\right)\;c_{\mathrm{sat}}\left(T\right), & \text{at }R=R_{\mathrm{out}}\;\left(\text{external}\right)\text{.} \end{array}$$

In (53), (54), *μ*_*w*,*c*_ = *μ*_*w*_(*c*_*c*_) and *μ*_*w*,*s*_ = *μ*_*w*_(*c*_*s*_) represent the chemical potential at the core side and the skin side of the interface, respectively, and *D*_*μ*_ regulates how fast the liquid mass flows through the interface; we have set it to a small number. *β*_*cav*_ and *β*_*air*_ are the mass transfer coefficients of the two boundary layers, *c*_*sat*_(*T*) is the saturated molar water vapour concentration (in [mol/m^3^]) (following the Tetens relation), *RH*_*ext*_ and *RH*_*cav*_ are the relative humidities of environment and the cavity. The water activities relate to:

(55) $$\begin{array}{lll} \mu_{w}=R_{\mathrm{gas}}\;T/\nu_{w}\;\log\left(a_{w,int}\right) & & \text{at }R=R_{in}\;\left(\text{cavity}\right)\text{,} \end{array}$$

(56) $$\begin{array}{lll} \mu_{w}=R_{\mathrm{gas}}\;T/\nu_{w}\;\log\left(a_{w,ext}\right) & & \text{at }R=R_{\mathrm{out}}\;\left(\text{external}\right). \end{array}$$

The mass transfer coefficients are *β*_*cav*_ = *D*_*vap*_/(*R*_*cav*_/5), and *β*_*air*_ = *h*_*air*_/*ρ*_*air*_*c*_*p*,*air*_ following the Lewis relation. *h*_*air*_ ≈ 20 W/m^2^. K for air velocities about 1 m/s. *RH*_*cav*_ = *p*_*vap*_/*p*_*sat*_(*T*) follows from the mass balance for the water vapour in the cavity, with *p*_*vap*_ the vapour pressure, and *p*_*sat*_ is the saturated vapour pressure. The vapour pressure enters the gas pressure:

(57) $$p_{\mathrm{gas}}=p_{\mathrm{vap}}+p_{\mathrm{air}},$$

and *p*_*air*_ follows Gay-Lussac:

(58) $$\begin{array}{lll} p_{\mathrm{air}}\;V_{\mathrm{gas}} & = & p_{\mathrm{air,0}}\;4\;\pi \;R_{in}^{3}/3, \end{array}$$

(59) $$\begin{array}{lll} V_{\mathrm{gas}} & = & \lambda_{\theta }^{3}\;4\;\pi \;R_{in}^{3}/3, \end{array}$$

(60) $$\begin{array}{lll} p_{\mathrm{vap}} & = & N_{\mathrm{vap}}R_{\mathrm{gas}}T/V_{\mathrm{gas}}. \end{array}$$

*N*_*vap*_ is the state variable for the mass balance of the cavity, written as a 0D balance equation, implemented in COMSOL as an ordinary differential equation (ODE):

(61) $$\partial_{t}N_{\mathrm{vap}}=J_{\mathrm{cav}}4\pi R_{in}^{2}\lambda_{\theta }^{2}$$

Finally, the complete weak formulation for the coupled problem writes as: find *u*, *p*, *c*_*c*_, *c*_*s*_ such that:

(62) $$W_{1}\left(\tilde{u},\tilde{p}\right)+W_{2}\left(\tilde{u},\tilde{p}\right)+W_{c}\left({\tilde{c}}_{c}\right)+W_{s}\left({\tilde{c}}_{s}\right)=0\;\forall \tilde{u},\tilde{p},{\tilde{c}}_{c},{\tilde{c}}_{s}$$

The problem is supplemented with initial conditions for both core and skin:

(63) $$\begin{array}{lll} \nu_{w}c_{c}\left(t=0\right) & = & \left(1-\varphi_{\mathrm{core}}/\lambda_{0}^{3}\right)\\ \nu_{w}c_{s}\left(t=0\right) & = & \left(1-\varphi_{\mathrm{skin}}/\lambda_{0}^{3}\right) \end{array}$$

## 3 Simulation results

### 3.1 Steady state solutions

**Fig. 3:**
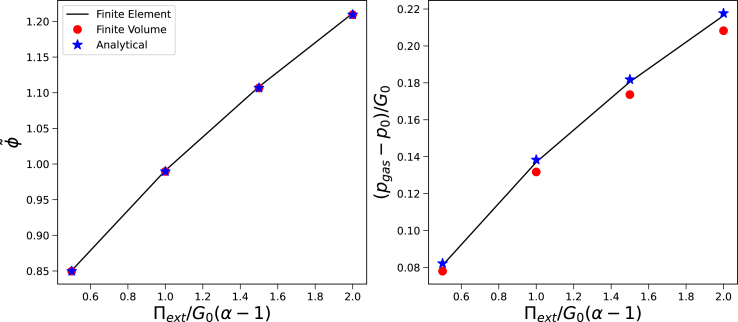
Steady state solution of a single domain system (*G*_*skin*_ = *G*_*core*_) regarding a) polymer volume fraction $\tilde{\varphi }$ (left pane) and b) pressure difference between cavity and environment *p*_*gas*_ − *p*_0_, as follows from the analytical solution, the Finite Element and Finite Volume method.

We first validate our simulation model against the analytical solution. We assume a single domain with *G*_*skin*_ = *G*_*core*_, subject the system to different external osmotic pressures Π_*ext*_. Simulations are performed for *R*_*out*_/*R*_*in*_ = 0.6 and *T* = 308 K. Results are shown in Fig. 3. We have scaled Π_*ext*_ against, (*α* − 1)*G*_*core*_ which is the osmotic pressure, where the gel would be in the unstretched state, $B_{u}$, with *λ*_*e*,*i*_ = 1 and *φ*_*s*_ = *φ*_*ref*_. The results show that both the Finite Element and the Finite Volume methods show good agreement with the analytical solution, but the Finite Volume method is less accurate in the predictions of the gas pressure, *p*_*gas*_.

**Fig. 4:**
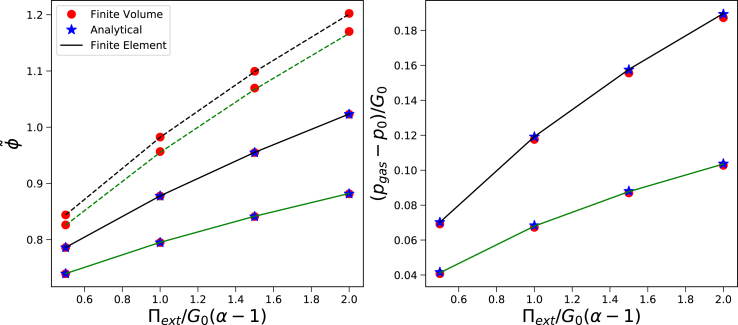
Steady state solution of a core/shell system (with either *G*_*skin*_ = 2*G*_*core*_ (black lines) or *G*_*skin*_ = 5*G*_*core*_ (green lines)) regarding a) polymer volume fraction $\tilde{\varphi }$ (left pane) and b) pressure difference between cavity and environment *p*_*gas*_ − *p*_0_, as follows from the analytical solution, the Finite Element and Finite Volume method.

Similar simulations are performed for the cases *G*_*skin*_/*G*_*core*_ = {2, 5}. Again, we have scaled Π_*ext*_ with (*α* − 1)*G*_*core*_, with *G*_*skin*_ = *G*_*core*_. As above, we have *R*_*out*_/*R*_*in*_ = 0.6 and *T* = 308 K. Results are shown in Fig. 4. Again, there is reasonably good agreement between the three solution methods. We take this result as a good validation of both the Finite Element and Finite Volume method.

**Fig. 5:**
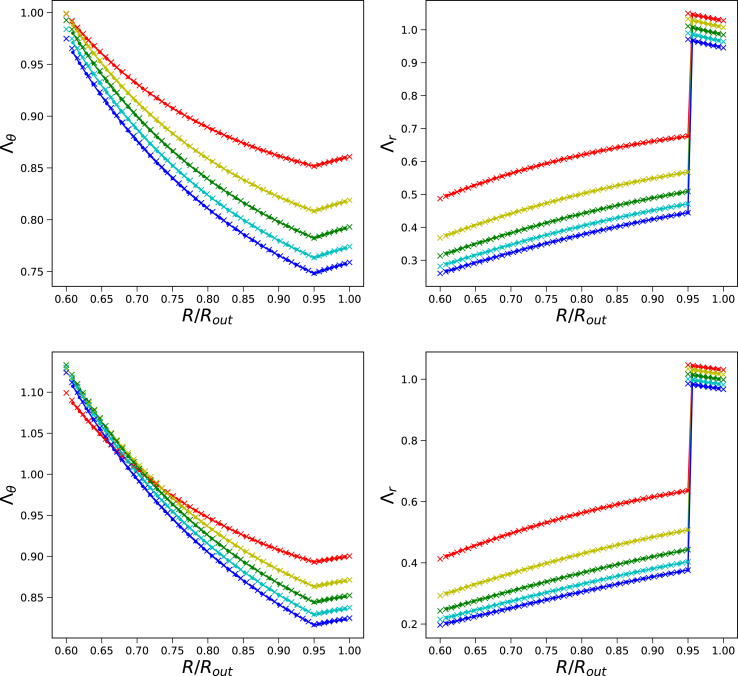
Steady state solution of a core/shell system (with *R*_*in*_/*R*_*out*_ = 0.6 and either *G*_*skin*_ = 10*G*_*core*_ (top panes) or *G*_*skin*_ = 20*G*_*core*_ (bottom panes) regarding *λ*_*θ*_ and *λ*_*r*_ for Π_*ext*_/*G*_*core*_ = {4, 8, 12, 16, 20} (rygcb) for the Finite Element (crosses) and Finite Volume method (lines).

We have performed a parameter study for *G*_*skin*_/*G*_*core*_ = {10, 20} and 4 ≤ Π_*ext*_/*G*_*core*_ ≤ 20, with results shown in Fig. 5. We show there the profiles of Λ_*θ*_ and Λ_*r*_ as a function of the position in the material frame *R*/*R*_*out*_. Observe the continuity in Λ_*θ*_, and the discontinuity in Λ_*r*_ at the core/shell interface. The solutions of the Finite Volume and Finite Element method coincide very well with each other. The results show that for *G*_*skin*_/*G*_*core*_ Λ_*θ*_(*R*_*in*_) = *r*_*in*_/*R*_*in*_ > 1 for all values of *Pi*_*ext*_, meaning that in the steady state the pore is larger than in the initial state. For *G*_1_/*G*_0_ = 10 the pore remains smaller than the initial pore size.

**Fig. 6:**
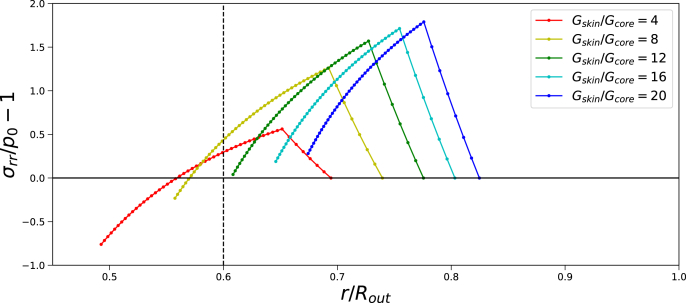
Radial stress *σ*_*rr*_ profiles (as function of current position *r*/*R*_*out*_) at steady state for different ratios of *G*_*skin*_/*G*_*core*_. Skin thickness is *t*_*skin*_ = 0.05*R*_*out*_, and the initial pore radius is *R*_*in*_ = 0.6*R*_*out*_, which is indicated with the dashed vertical line. If −*σ*_*rr*_(*r*_*in*_)/*p*_0_ − 1 < 0 the pore has expanded beyond its initial size. This condition is indicated by the solid horizontal line.

We investigate the pore growth via a parameter study for Π_*ext*_/*G*_0_ = 20, while varying *G*_*skin*_/*G*_*core*_ to indicate a critical ratio for pore opening. Results are shown in Fig. 6. We observe that for *G*_*skin*_/*G*_*core*_ > 10 it occurs that *p*_*gas*_ = −*σ*_*rr*_(*r*_*in*_) < *p*_0_, meaning that the pore has expanded beyond its initial size *R*_*in*_. This is also observed for the actual position of the pore for *G*_*skin*_/*G*_*core*_ > 10, which is right of the vertical dashed line indicating the initial pore size.

**Fig. 7:**
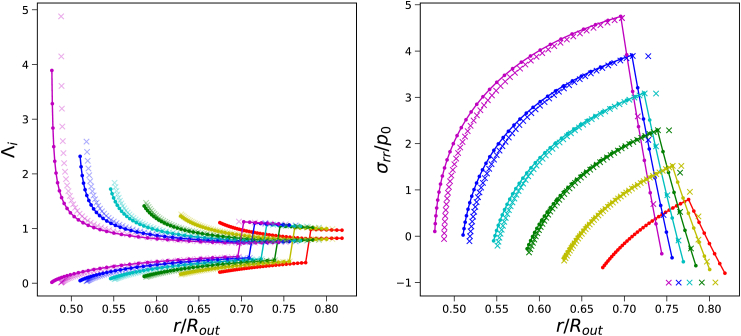
Principal stretches *λ*_*θ*_ and *λ*_*r*_ (left pane), and stress profiles *σ*_*rr*_ as function of current position *r*/*R*_*out*_ at steady state for different ratios of *R*_*in*_/*R*_*out*_. Skin thickness is *t*_*skin*_ = 0.05*R*_*out*_, and *G*_*skin*_/*G*_*core*_ = 20 and Π_*ext*_/*G*_*core*_ = 20. The Finite Volume solution is indicated with solid lines(with dots), and the Finite Element solution is indicated with crosses.

We investigate the development of gas pressure as a function of initial pore size, using Π_*ext*_/*G*_*core*_ = 20, *G*_*skin*_/*G*_*core*_ = 20, and *t*_*skin*_ = 0.05*R*_*out*_. First, we analyse the principal stretches and stress profiles, which are shown in Fig. 7. We observe that for smaller pores, stronger gradients in *σ*_*rr*_ and Λ_*θ*_ develop at the interface between the core and gas bubble. Finite Volume and Finite Element solutions are quite similar. Additional simulations show that Finite Volume simulations converge to the Finite Element solutions with the increase of the number of grid points. However, Finite Volume simulation for the smallest pore becomes unstable.

**Fig. 8:**
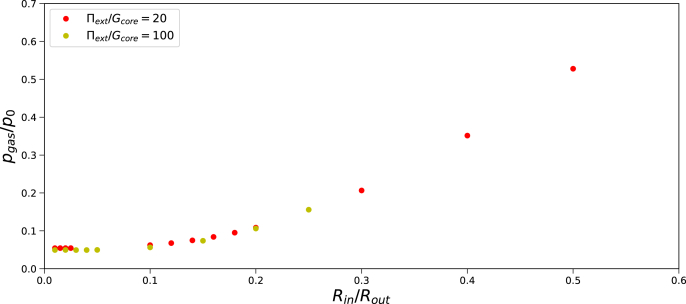
Gas pressure *p*_*gas*_/*p*_0_ as function of initial pore radius *R*_*in*_ for Π_*ext*_/*G*_*core*_ = 20, *G*_*skin*_/*G*_*core*_ = 20, and *t*_*skin*_ = 0.05*R*_*out*_ following the Finite Element solution.

Gas pressure as a function of pore radius is shown in Fig. 8, as obtained from Finite Element simulations. We observe that with decreasing pore radius, *R*_*in*_, the gas pressure approaches a limiting value, which is just above zero. Hence, there is no sign of instability here. We investigate further the case of Π_*ext*_/*G*_*core*_ = 100, which shows similar behaviour, albeit a slightly lower final pressure. The limiting pressure is about reached if *R*_*in*_/*R*_*out*_ ≈ 0.05.

**Fig. 9:**
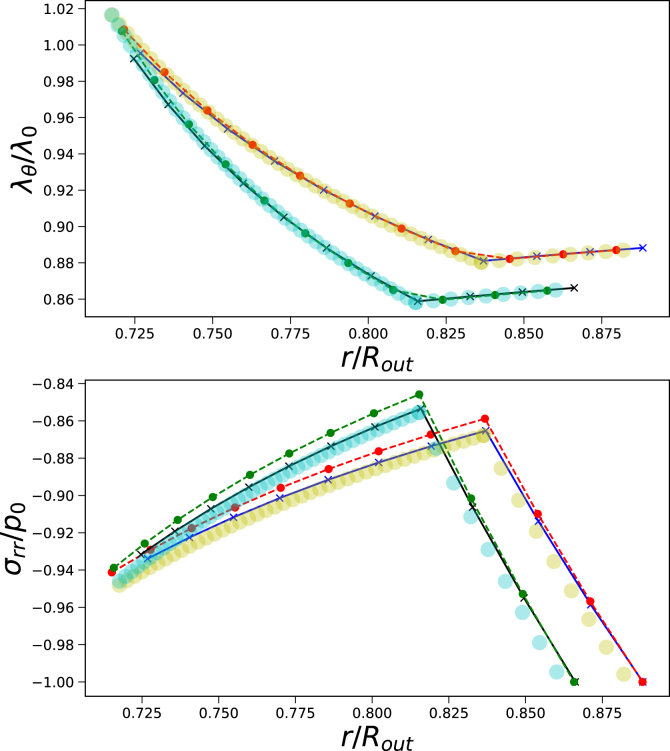
Comparison of steady-state solution (*λ*_*θ*_ and *σ*_*rr*_) according to a) Finite Element solver (translucent symbols), b) Finite Volume steady state solver (solid lines), and c) Finite Volume transient solver (dashed lines). Simulations are performed for *G*_*skin*_/*G*_*core*_ = 20, *t*_*skin*_ = 0.05*R*_*out*_, *R*_*in*_ = 0.70*R*_*out*_, and two values of Π_*ext*_/*G*_*core*_ = {6, 10} (indicated by yellow and cyan symbols respectively).

We investigate the performance of the transient Finite Volume solver. We compare its steady-state solution with those of the Finite Volume steady-state solver (using similar discretization), and the Finite Element solver. Simulations are performed for *G*_*skin*_/*G*_*core*_ = 20, *t*_*skin*_/*R*_*out*_ = 0.05, *R*_*in*_/*R*_*out*_ = 0.7, and Π_*ext*_/*G*_*core*_ = {6, 10}. These values were chosen to keep the computation time of the transient Finite Volume solver small. Results are shown in Fig. 9. We conclude that the three solvers give quite comparable solutions for *σ*_*rr*_, but they are in good agreement for *λ*_*θ*_.

**Fig. 10:**
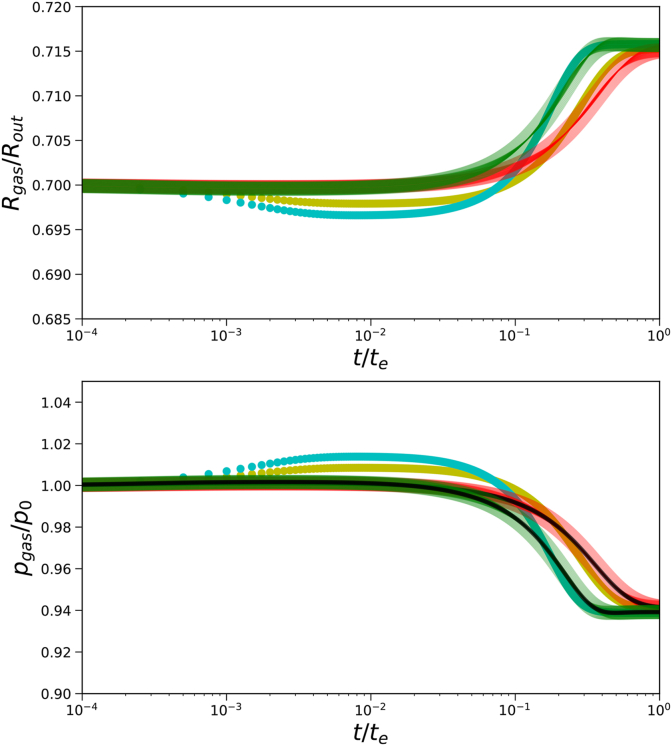
Comparison of transient solutions of gas pressure *p*_*gas*_ and pore size *R*_*gas*_ according to a) Finite Element solver (translucent symbols), b) analytical solution (black lines), and c) Finite Volume transient solver (yellow and cyan solid lines). Simulations are performed for *G*_*skin*_/*G*_*core*_ = 20, *t*_*skin*_ = 0.05*R*_*out*_, *R*_*in*_ = 0.70*R*_*out*_, and two values of Π_*ext*_/*G*_*core*_ = {6, 10} (indicated by yellow and cyan symbols respectively).

Furthermore, we have compared the transient solution of the gas pressure and pore radius for the Finite Volume and Finite Element solvers, as shown in Fig. 10. Due to some differences in the boundary condition at the outer boundary, there is some difference in the dynamics, but overall there is similar behaviour. Both solutions show an equal expansion of the pore. Surprisingly, the Finite Element solution for the gas pressure follows exactly the analytical solution, Eq. (34), as indicated by the black lines. The mass transfer is so slow, that the gas pressure follows a quasi-steady state solution.

The (transient) Finite Element solver has a much shorter computation time than the transient Finite Volume solver, as the latter was based on Python, which is interpreter-based. We assume all solvers to be valid, but given the difference in computation time, further analysis is done with the Finite Element solver.

**Fig. 11:**
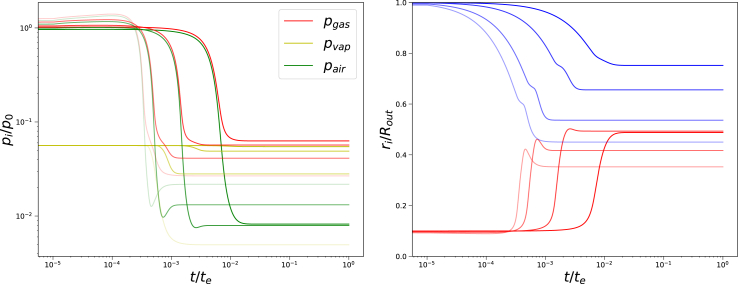
Left pane: Gas, and partial vapour and air pressures as a function of time, and rightpane: inner (red) and outer radius (blue) of the system as a function of time. Both graphs as from simulations with *G*_*skin*_/*G*_*core*_ = 20, *t*_*skin*_ = 0.05*R*_*out*_, *R*_*in*_ = 0.1*R*_*out*_, Π_*ext*_/*G*_*core*_ = {20, 100, 500, 1750} (decreasing order of transparency) following the Finite Element solution.

First, we investigated the evolution of the total and partial gas pressures for a range of external pressures Π_*ext*_/*G*_*core*_ = {20, 100, 500, 1750}, as shown in Fig. 11. At very short times we observe compression of the central pore, shown by the increase of gas and air pressure. Subsequently, we observe that first, the partial air pressure decreases due to the instantaneous increase of pore size, because the drying front reaches the central pore. Only then the partial vapour pressure decreases, which remains in equilibrium with the hydrogel bounding the pore. At large values of the external pressure Π_*ext*_/*G*_*core*_ ≥ 100 the air pressure goes through a minimum, before stabilizing at a larger value. Remarkably, the final air pressure exceeds the final vapour pressure at the maximal external pressures Π_*ext*_/*G*_*core*_ = 1750. The Finite Element solver is unstable for Π_*ext*_/*G*_*core*_ ≥ 2000, possibly due to physical instability of the central cavity.

In the right pane, one can also observe the evolution of the gel radius *r*_*out*_ and pore radius *r*_*in*_ with time. Similar to the air pressure, we observe a temporary extremum in the pore radius. This extremum in the radius is explained by the sudden expansion of the pore, which happens faster than the diffusion of moisture in the core. Consequently, it takes time for the vapour pressure to reach a new equilibrium, as shown in the graph for the vapour pressure *p*_*vap*_ in the left pane. Due to this extra supplementation of total gas pressure, the radius shrinks back for a small amount.

Furthermore, the sudden increase of *r*_*in*_ is faster for increasing external osmotic pressure Π_*ext*_. The difference between final values of *r*_*in*_ and *r*_*out*_ becomes smaller with increasing Π_*ext*_, but their ratio goes to an asymptote.

**Fig. 12:**
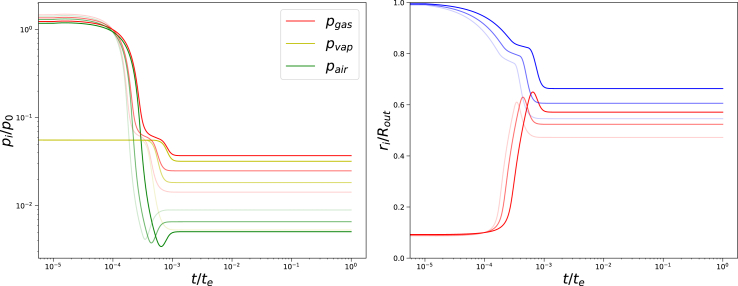
Left pane: Gas, and partial vapour and air pressures as a function of time, and right pane: inner (red) and outer radius (blue) of the system as a function of time. Both graphs as from simulations with *T* = 308*K* (35^*o*^*C*), *G*_*skin*_/*G*_*core*_ = 50, *t*_*skin*_ = 0.05*R*_*out*_, *R*_*in*_ = 0.1*R*_*out*_, Π_*ext*_/*G*_*core*_ = {400, 800, 1800} (decreasing order of transparency) following the Finite Element solution.

**Fig. 13:**
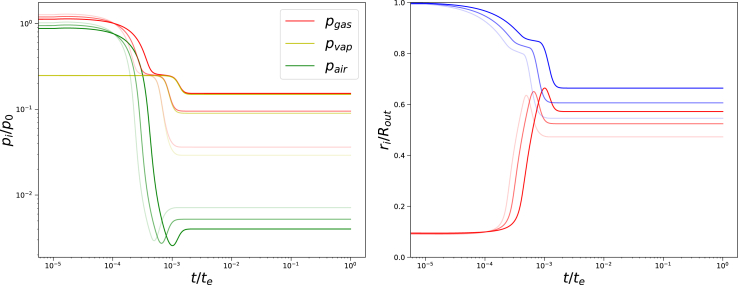
Left pane: Gas, and partial vapour and air pressures as a function of time, and right pane: inner (red) and outer radius (blue) of the system as a function of time. Both graphs as from simulations with elevated temperature *T* = 338*K* (65^*o*^C), and *G*_*skin*_/*G*_*core*_ = 50, *t*_*skin*_ = 0.05*R*_*out*_, *R*_*in*_ = 0.1*R*_*out*_, Π_*ext*_/*G*_*core*_ = {400, 800, 1800} (decreasing order of transparency) following the Finite Element solution.

We perform a similar analysis for a higher ratio *G*_*skin*_/*G*_*core*_ = 50. Results are shown in Fig. 12. We observe qualitatively similar behaviour as in the case of *G*_*skin*_/*G*_*core*_ = 20. However, the shrinkage of the outer radius is much less, and the pore opening is larger. The instability of the code happens at similar conditions Π_*ext*_/*G*_*core*_ ∼ 2000. We have also performed similar simulations at higher temperatures, which should give higher vapour pressures. We have changed temperature to *T* = 338*K* (65^*o*^*C*), with *G*_*skin*_/*G*_*core*_ = 50. Results are shown in Fig. 13. Again results are qualitatively similar, albeit with higher vapour pressures. Total gas pressure is mainly determined by the vapour pressure. However, the instability of the scheme is still a similar condition: Π_*ext*_/*G*_*core*_ ∼ 2000. Investigation of how final gas pressures depend on Π_*ext*_ does not show any indication of an onset of physical instability. Hence, it is likely that the instability is numerical.

Calculations show that the core material will have *a*_*w*_ ≈ 0.07 in case of Π_*ext*_/*G*_*core*_ ∼ 2000. At this low water activity at the end of drying most food biopolymers will be in the glassy state, and they will have a high elastic modulus (Van der Sman et al., 2022) (van der Sman et al., 2023), as is also indicated in our discussion section. Hence, our approximation of a constant elastic modulus of the core material is not realistic. Consequently, we think it is not worthwhile to resolve the apparent numerical instability.

## 4 Discussion

In this paper, we have shown that substantial pore formation and growth can occur during the drying of soft materials in the presence of a stiff elastic skin. The transient and steady-state solutions of the Finite Volume solver compare quite well with the Finite Element solutions. In the case of relatively large pores, the steady-state solutions also compare well with the analytical solutions. If pore formation is gradual, the gas pressure in the pore also follows the analytical solution - showing that the pore is in a quasi-steady state.

The validity of the analytical solution is restricted to cases where the polymer volume fraction remains relatively uniform in both skin and core materials. The Finite Element solver has a significantly wider range of numerical stability than the Finite Volume solver. However, a Finite Volume implementation can still have its merits, because they are much easier to implement in general-purpose programming languages like C/C++ and Python. Also, they might be more suited in multiscale simulation, where pore formation is solved at the mesoscale, which is coupled to heat/mass transfer at the macroscale - similar to the multiscale cell model for the expansion of starchy snacks (van der Sman and Broeze, 2014a, 2014b).

We have also investigated the physics of the pore formation in more detail - bas shown in Fig. 11, Fig. 12, Fig. 13. In the absence of an elastic skin, we find that the initial pore only undergoes shrinkage. Only, if the skin has sufficient stiffness, *G*_*skin*_/*G*_*core*_ > 10, one observes that the pore expands. The smaller the initial pore size, the lower the final gas pressure. The relative growth of the pore *r*_*in*_/*R*_*in*_ increases with decreasing initial pore size. The pore expansion problem also has interesting dynamics: after an initial pore compression stage, the pore suddenly expands - which reaches a maximum just before the drying front reaches the inner pore. This moment of drying front reaching the inner pore is indicated by the fact that the vapour pressure reaches the equilibrium value, as determined by the external osmotic pressure Π_*ext*_.

In our simulations, we have not observed any physical instability, where the pore expands without bounds. There is a regime where the Finite Element scheme becomes unstable, but extrapolations of gas pressure and pore size into this regime do not hint at the case of physical instability but numerical instability. This numerical instability occurs at relatively extreme conditions, Π_*ext*_/*G*_*core*_ ≈ 2000, meaning that the soft material is quite dry (*a*_*w*_ < 0.1). Food materials are commonly in the glassy state, and our approximation of the core material as a hydrogel with a constant elastic modulus does not hold anymore. Consequently, we think that the Finite Element solver is well applicable to problems of vegetable and fruit drying. The Finite Element model is also tested at elevated drying temperature, which renders elevated vapour pressures, which thus enhance the total gas pressure in the expanding pore. With increasing temperature, we observe an increase in the ratio of *r*_*in*_/*R*_*out*_, which means an increase in porosity.

For our model to approach the realistic food material/vegetable drying process, we need to account for their viscoelastic properties (Ozturk and Takhar, 2020) (Hu et al., 2023), and the change of such properties with temperature and moisture content. Fundamental theories for their viscoelastic properties still need to be developed. However, for proteins we have shown that their elastic modulus changes with *T*_*g*_/*T*, the ratio of the moisture-dependent glass transition temperature and the actual temperature (van der Sman et al., 2023), and for starch and maltodextrins we have shown that zero-shear viscosity and relaxation times also scale with *T*_*g*_/*T* (Van der Sman et al., 2022). Accounting for viscoelasticity is important for modelling food drying, as it explains hysteresis of moisture sorption (Meinders and Oliver, 2015) (van der Sman, 2023) (Hu et al., 2023), and thus that the elastic stresses need to be included in the driving force for moisture transport, as also shown in this paper. The increase of elastic modulus and relaxation times with decreasing moisture content explains the formation of a rigid skin/case hardening during drying, despite starting with a material fully in the rubbery state at the start of drying. At sufficient progression of drying the relaxation times for the core tissue become so large that elastic stresses are not relaxed away, and an increase of porosity is observed (Nguyen et al., 2018). Extension of the current model of the spherical system with pore towards viscoelastic properties is presented in a forthcoming paper (van der Sman et al., 2024).

Further extension of the model is envisaged towards expansion of food materials during intensive heating as baking or frying. This requires the coupling of the viscoelastic model towards an energy balance, and making the material properties temperature dependent. In previous papers we have shown that viscoelastic properties of starches and proteins scale with *T*_*g*_/*T*, the ratio between glass transition and actual temperature. Near and beyond the glass transition, food materials behave as elastic material with very long viscoelastic relaxation times. Prelimenary simulations show strong expansion of the pore leading to increase of the outer radius of the spherical system beyond its initial size. Preconditions for the strong expansion are: a) initial conditions are near the glass transition, and b) the product temperature needs to reach conditions near or beyond the boiling point of water - which renders gas pressures larger than atmospheric pressure.

## 5 Conclusion

In this paper, we have shown a physical model that can explain the increase of porosity in food materials during drying. A prerequisite for this phenomenon is the presence of an elastic skin, having a significantly higher elastic modulus than the core material. In our model, we included a pre-existing pore filled with air and water vapour, which gets enlarged due to tensile stresses induced by the hindered shrinkage of the elastic skin and the continued moisture removal from the core via the drying. The moisture transport and large deformation mechanics are strongly coupled via the pressure field entering both the stress and the chemical potential.

A previously developed Finite Volume implementation has been validated against a (COMSOL) Finite Element implementation and an analytical solution. The Finite Element solution is numerically stable over a wider range of parameters. The Finite Element model shows stable pore growth even under severe drying conditions resulting in low water activities *a*_*w*_ ≈ 0.1, and elevated temperatures. Like similar models applied to dehydrating hydrogels, the Finite Element model is formulated via the weak form, which is mathematical rather intricate. Because of the rare use of the weak form in food science, we have explained in a tutorial manner how to derive this weak form. We think only this kind of modelling with a multi-physical coupling of large deformations, heat, and mass transfer can explain the microstructural changes in food material during drying, and intensive heating processes. With the latter, we think of the creation of porous foods via baking or frying. The next step in making our model more realistic is the inclusion of temperature, and moisture-dependent viscoelastic properties.

## CRediT authorship contribution statement

**R.G.M. van der Sman:** Conceptualization, Methodology, Software, Investigation, Writing – original draft, Writing – review & editing. **Michele Curatolo:** Methodology, Software. **Luciano Teresi:** Methodology, Software, Writing – review & editing.

## Declaration of competing interest

Authors declare there is no conflict of interest.

## Data Availability

No data was used for the research described in the article.

## Appendix Derivation of the Weak Form

We derive the weak form for time-dependent elliptical equations, such as our mass balance. The strong form is (in spherical coordinates):

(64) $$\partial_{t}c-M\partial_{R}^{2}\mu_{w}=0$$

Multiplication with test function $\tilde{c}$, and integration over the domain renders:

(65) $$\int \left(\tilde{c}\partial_{t}c-M\tilde{c}\partial_{R}^{2}\mu_{w}\right)4\pi R^{2}dR=0$$

Note that:

(66) $$\partial_{R}\tilde{c}MR^{2}\partial_{R}\mu_{w}=\tilde{c}\partial_{R}\left(MR^{2}\partial_{R}\mu_{w}\right)+MR^{2}\partial_{R}\mu_{w}\partial_{R}\tilde{c}$$

And hence, the final weak form is:

(67) $$\int \left(\tilde{c}\partial_{t}c+M\partial_{R}\tilde{c}\partial_{R}\mu_{w}\right)4\pi R^{2}dR={\left[\left(4\pi R^{2}\tilde{c}M\partial_{R}\mu_{w}\right)\right]}_{R_{2}}^{R_{1}}$$

The right-hand side will be implemented as boundary conditions. The left-hand side is the weak form programmed in COMSOL as:

(68) $$4\pi R^{2}\left(test\left(c\right)*ct-test\left(cR\right)*Nw\right)$$

with Nw = *M∂*_*R*_*μ*_*w*_ the molar flux of water (in mol/m^2^/s).

We derive the weak formulation for the momentum balance for spherical coordinates. We start with the strong form (∇ ⋅**S** = 0):

(69) $$\partial_{R}S_{RR}+\frac{2}{R}\left(S_{RR}-S_{\Theta \Theta }\right)=0$$

Integrate it over the volume *R*_1_ < *R* < *R*_2_, with test function $\tilde{u}$:

(70) $$\int_{R_{1}}^{R_{2}}\left[\partial_{R}S_{RR}+\frac{2}{R}\left(S_{RR}-S_{\Theta \Theta }\right)\right]4\pi R^{2}\tilde{u}dR=0$$

Integration by parts is applied to the first term only:

(71) $$\int_{R_{1}}^{R_{2}}-4\pi R^{2}\left[S_{RR}\left(\partial_{R}\tilde{u}\right)+\frac{2}{R}S_{RR}\tilde{u}\right]+\frac{2}{R}\left(S_{RR}-S_{\Theta \Theta }\right)4\pi R^{2}\tilde{u}dR=b.c.$$

which finally renders:

(72) $$\int_{R_{1}}^{R_{2}}-\left[S_{RR}\left(\partial_{R}\tilde{u}\right)+\frac{2}{R}S_{\Theta \Theta }\tilde{u}\right]4\pi R^{2}dR=b.c.$$

In COMSOL the weak formulation of the momentum balance reads:

(73) $$4\pi R^{2}\left[-test\left(uR\right)\;Srr-\frac{2}{R}Sqq\;test\left(u\right)\right]$$

*u* is the displacement in the radial direction. *R* is the coordinate in the domain frame. *uR* = *∂u*/*∂R*. $Srr=S_{RR}$ and $Sqq=S_{\Theta \Theta }$.
